# Health and Social Care Diversity Among Individuals with Longstanding Physical and Psychological Health Problems: Pooled Repeated Cross Sectional Analyses

**DOI:** 10.1007/s10597-020-00566-y

**Published:** 2020-02-08

**Authors:** Corine Driessens, David Kingdon, David Pilgrim, Peter W. F. Smith

**Affiliations:** 1 Faculty of Medicine, Clinical and Experimental Science; Room 83 Level F, South Academic Block, Southampton General Hospital, University of Southampton, Southampton, SO16 6YD England, UK; 2 Department of Psychiatry Academic Centre, University of Southampton, College Keep Room 123, 4 - 12 Terminus Terrace, Southampton, SO14 3DT England, UK; 3 Department of Clinical Psychology, University of Southampton, Highfield Campus, Southampton, SO17 1BJ England, UK; 4 Department of Social Statistics & Demography - Social Sciences, University of Southampton, Building 39 Room 2009, Southampton, SO17 1BJ England, UK

**Keywords:** Health care, Social care, General household survey, General lifestyle survey, Mental health problems, Parity of esteem

## Abstract

This paper examines differences in health-and-social care utilisation for individuals with physical and/or mental health problems. Logistic regression models are used to determine disparity in the percentage of General Household/Lifestyle Survey participants with physical compared to mental health problems receiving disability benefits or health care services between 2000 and 2011. Our findings of a relative underutilisation of secondary health care combined with a relative overutilization of out-of-work benefits by individuals with mental health problems is novel to the field of rehabilitative health care. These results provide evidence for the previously suspected disparity in health care utilisation of individuals with mental health problems and indicate problems in labour force integration. The findings support the political call for a ‘parity of esteem’, which, in Britain, was enshrined in the Health and Social Care Act of 2012.

**Electronic supplementary material:**

The online version of this article (10.1007/s10597-020-00566-y) contains supplementary material, which is available to authorized users.

## Introduction

In Britain, 17% of the working-age adults live with mental health problems (Bebbington and McManus 2019) and only one in three of these adults receive treatment for their mental health problems (McManus et al. 2016). The mortality of people with mental problems living in the community is 1.9 times higher than the mortality rate of the community population without mental health problems (Walker et al. 2015). Factors in the healthcare system contributing to this increase include side effects or non-collaboration with psychiatric medication, limited access and fragmentation of health services, as well as poor quality of physical health services (Firth et al. 2019; Liu et al. 2017).

Between 1995 and 2014, there was an increase of individuals with mental health problems and a decrease of individuals with physical health problems claiming out-of-work disability benefits (Viola and Moncrieff 2016). In 2004 there were more incapacity benefits claimants with mental health problems than there were unemployed individuals receiving jobseekers’ allowance (Layard 2005). Although the government restricted public spending on disability this did not affect the number of out-of-work disability benefits claimants with mental health problems. The Labour Force Survey data shows an increase in the people reporting mental health problems since the recession in 2008, especially among people who were out of work and people with lower levels of education (Barr et al. 2015).

The above literature describes diversity in the health and social care received by individuals with physical and mental health problems. In this study we investigate the nature of this inequality for British individuals reporting longstanding illness/disability/infirmity. Data is taken from the General Household Survey [GHS]/General Lifestyle Survey [GLS] between 2000 and 2011 to examine the differences in health care services provided by primary, secondary, or emergency health care professionals and social care received for disability by individuals reporting physical compared to mental longstanding illness/disability/infirmity taking into account how severely daily activities were limited by these health issues.

## Methods

### Sample

This study is a secondary analysis of data collected as part of the GHS and GLS between 2000 and 2011. Each year, the Social Service Division of the Office for National Statistics (ONS) collected data from 18,000 to 30,000 individuals living in 13,000 private households. The GHS was last run in 2007 and its questions were incorporated into a larger enterprise known as the GLS. Response rate to the GHS/GLS ranged between 67 and 76%.

Before 2005, a different sample of households was picked annually using a stratified sampling design to improve population representativeness. In 2005, a longitudinal design was adopted. Participants remained in the sample for 4 years with one quarter of the sample being replaced each year. To remove potential source of dependence between data from different waves, we focused only on first wave participants.

More specifically, we focused on the participants classified within this first wave by the ONS as having a physical health problem, a mental health problem, or a physical-and-mental health problem based on their response to the questions:

“Do you have any longstanding illness, disability, or infirmity?”

“What is the matter with you?”

“Does this illness or disability limit your activity in any way?”

Between 21.9 and 28.8% of the participants included in the GHS/GLS disclosed having longstanding physical health problems, 1.5–2.6% detailed having longstanding mental health problems, and 0.8–1.8% described having longstanding physical & mental health problems.

Individuals living in institutions were not included in the GHS/GLS. Since individuals age > 65 with mental health problems were more likely to be institutionalized, the true population mental health rate for the older population in the GHS/GLS was under-estimated. We limited our sample to participants aged 16–64, excluding yearly between 36.3 and 38% of the individuals. This percentage was equivalent to the age dependency ratio quoted in the ‘Overview of UK population: March 2017’ published online by ONS.

### Health Care

The questions in the health section on the GHS/GLS remained unchanged between 2000 and 2011:

“During the 2 weeks ending yesterday, apart from any visit to a hospital, did you talk to a doctor for any reason at all, either in person or by telephone?”

“On whose behalf was this consultation made?”

“Was the doctor a General Practitioner (GP)/family doctor?”

“During the last 2 weeks ending yesterday, did you see a practice nurse at the GP surgery on your own behalf?”

“During the last 3 months did you attend as a patient at the casualty or outpatient department of a hospital (apart from straightforward ante- or post-natal visits)?”

“Apart from maternity stays, have you, in the past year, been in hospital for treatment as a day patient, i.e. admitted to a hospital bed or day ward, but not required to remain overnight?”

“Apart from maternity stays, have you, in the past year, been in hospital as an inpatient, overnight or longer?”

Information gathered with these questions was used to create the variables: Seen nurse, Seen GP, Emergency care, Outpatient Care, Daypatient care, and Inpatient care. Non-response to these questions on receipt of primary and secondary health care services was limited to 2.8%.

### Social Benefits

A vast array of disability benefits were available to disabled individuals living in Britain between 2000 and 2011 (Rowlingson and Berthoud 1996; Spicker 2011). For these analyses these disability benefits were divided into the following categories:

- benefits to compensate people for injury at work: industry injury, war disability, war widow
- benefits to meet extra costs of living as a disabled person: care component Disability Living Allowance (DLA), mobility component DLA, attendance allowance
- in-work benefits for disabled people: disability working allowance, (family) work tax credit, back to work
- earnings-replacement benefits for those deemed incapable to work: incapacity benefit, severe disablement allowance, income support

In 2001 severe disablement allowance was replaced by incapacity benefit. However, those individuals already receiving severe disablement allowance continued to do so to date. Incapacity benefit was phased out after the welfare reform act of 2007, but compulsory reassessment did not start until 2011, when GHS/GLS was discontinued. Since the GHS/GLS participants reported longstanding disabilities we assumed that they were still receiving incapacity benefits between 2007 and 2011. In 2008 income support was replaced by employment and support allowance, a benefit not measured by GHS/GLS.

### Secure Data

The secure version of the GHS/GLS data analysed during the current study is available upon request from the ONS and can be accessed within their secure datalab. Information on health status was protected under the Data Protection Act. Although the data for this study was anonymized, the health status of the anonymized GHS/GLS participants was known to the researcher. To take precautionary measures against disclosure of this sensitive personal information the analyses were preformed within the Secure Research Service (SRS) facility of the ONS. Only trained researchers accessed the data. Data was not released into the public domains unless thoroughly checked that it did not pose a risk of disclosure by 2 officers from the ONS. When a cell count was less than 10 disclosure risk was deemed too high.

### Statistics

This study encompassed secondary analyses of data collected during the first wave GHS/GLS interviews from 2000 to 2011. The analyses focused on the percentage of individuals receiving health and social care. Logistic regression was used to describe the nature of potential associations and to determine if the relationship persisted when controlling for time and potential confounding variables. GHS/GLS was a household survey, a random effect was therefore used to control for dependence among household members responses (Allison 2012). The unweighted data posed a disclosure risk when analysing the yearly waves separately. Hence, the data from all 12 years was pooled into 1 dataset.

### Regression with Pooled Cross-Sectional Data

By pooling the data we gained sample size which increased the precision of the estimators. However, it was important to consider whether the same logistic regression model applied to the data from each time period (Wendt 2007; Thomas and Wannell 2009). Pooled data was assumed to be a random sample from a population over time. Thus, in order to pool the data from the GHS/GLS, annual weights, were applied in order to make each annual sample representative of the British population (Firebaugh 1997). These annual weights had been created by the ONS for the GHS/GLS data from 2000 onwards.

We determined disparity in health and social care utilisation for individuals reporting physical compared to mental health problems. The models were separated depending on the limitations experienced by the participants: no limitations, somewhat limited by health problems; strong limitations due to health problems. If the aggregated outcome variable had changed over time, a time dummy variable was added to the logistic regression model.

Slightly more participants reported a health problem in 2002 and slightly less participants reported a health problem in 2003, 2008, 2009, and 2010. However, the balance between people reporting physical versus mental health problems remained the same during this period. Except for 2010 and 2011 when people with physical health problems were slightly more likely to also report mental health problems. There were slightly more participants reporting limitations in 2002 and 2003 than the other years.

Based on the limited flux in the populations of individuals with physical and mental health problems from 2000 to 2011, we determined that the relationship we were estimating remained stable over time.

### Confounding Variables

Prior research showed age, gender, ethnic, employment, and socio-economic differences in health and social care utilisation (Twomey et al 2015; Berthoud 2011). To control for the confounding effects of these variables on health and social care utilisation we included age (16–44 versus 45–64), gender, work status (not working/working), and ethnicity (white versus non-white) into the logistic regression model. Since health care inequalities differed when assessed with different measures of socioeconomic status (McCartney et al. 2017) various different socio-economic indicators were taken into account (renting/owning housing, owning computer, owning car, job level) in the analyses.

### Patient and Public Involvement

The study presented in this paper was based on a sample of individuals with health problems from the general British population. It included only 30–35% of the individuals included in the General Household/Lifestyle Survey. On request of several government departments the ONS interviewed individuals living in private households in Britain. The data was mainly use for government reporting and analysis, commissioning, service planning, performance management, and policies/legislation. Occasionally the data was requested for specific scientific use, disseminated to a peer-reviewed journal, and presented at scientific conferences and seminars. Ethical approval for the work presented in this manuscript was obtained from the Ethics and Research Governance of the University of Southampton (ERGO-19537).

All authors certify responsibility for the manuscript and state that there are no competing interests to declare.

## Results

### The Sample

27,846 individuals (weighted sample size 70,328,017) reported a health problem between 2000 and 2011. Participant characteristics and socioeconomic status were comparable to the British population at the time of the data collection (Table 1). Computer ownership increased over the years from about 60% in 2000 to 90% in 2011. Some form of health care services were received by 50.9% of the disabled individuals and 27.9% received some form of disability benefit. Despite pooling the data the sample size for benefits due to injury at work was so small that there was a disclosure risk, hence, no modelling was done with this group.

**Table 1:** Description of the sample

	Limitations to Activities			Overall sample
	None %	Some %	Strong %	%
Percentage of sample	44.5	31.1	24.3	100
Illness/disability/infirmity				
Physical	94.6	88.6	85.8	90.5
Mental	4.1	7.2	6.5	5.7
Physical & mental	1.3	4.2	7.7	3.8
Female	50.4	51.8	58.0	52.6
Age				
16–44	46.1	41.1	36.8	42.4
45–64	53.9	58.9	63.2	57.6
Caucasian	93.6	92.7	92.2	92.9
North England	44.7	47.1	50.1	46.7
South England	55.3	52.9	49.9	53.3
*Socio-economic characteristics*				
Working status				
Full-time/part-time	76.8	52.2	39.2	59.7
Not working	23.2	47.8	60.8	40.3
Not working past 12 months	5.9	12.8	17.4	15.5
Own Car	87.7	78.6	76.3	82.1
Own House	84.7	84.9	87.4	85.2
Own Computer	76.0	66.3	64.3	70.2
Job level				
Routine/manual	38.9	48.4	50.7	44.7
Intermediate	22.1	22.9	20.7	22
Managerial/professional	39.1	28.7	28.6	33.3
*Social care*				
Out of work	4.0	23.8	37.1	18.3
Extra-cost of living	2.3	15.2	24.2	11.8
In-work	3.9	3.5	3.2	3.6
Compensation for injury at work	.9	3.0	5.2	2.6
*Health care*				
Seen nurse	7.9	8.3	12.5	9.2
Seen GP	16.6	20.1	38.2	22.8
Emergency care	4.2	5.0	8.3	5.5
Outpatient care	12.9	20.8	31.3	20.2
Daypatient care	8.7	13.0	18.4	12.4
Inpatient care	6.4	11.7	20.7	11.6

### Differences in Health and Social Care

In this study, we ran different logistic regression models depending on the limitations caused by the health problems experienced (Tables 2, 3, 4) to determine if morbidity also contributed to inequalities to health and social care utilisation above and beyond the impact of the previously reported participant and socio-economic factors and found that significantly more individuals with mental health and physical & mental health problems received out of work benefits between 2000 and 2011 compared to individuals with just physical health problems. Significantly more individuals not limited by their mental health problems received GP care, in-work benefits, and extra-cost of living benefits compared to individuals not limited by just physical health problems. On the other hand, significantly more individuals not limited by their physical & mental health problems between 2000 and 2011 received day-patient and emergency care as well as extra-cost of living benefits compared to individuals not limited by just their physical health problems. Significantly less individuals limited by their mental health problems received outpatient care, day-patient visits, and extra-cost of living benefits compared to individuals limited by just their physical health problems. Significantly more individuals somewhat limited by their physical & mental health problems received GP and emergency care compared to individuals somewhat limited by just physical health problems. Also, significantly less individuals strongly limited by their mental health problems received inpatient care compared to individuals strongly limited by just their physical health problems between 2000 and 2011.

**Table 2:** Primary and emergency care of disabled individuals

	Nurse			General practitioner			Emergency care		
	Limitations			Limitations			Limitations		
	None N = 12,292	Some N = 8554	Strong N = 5982	None N = 11,999	Some N = 8281	Strong N = 6393	None N = 12,290	Some N = 8595	Strong N = 6706
Mental	.73 (.48–1.11)	.92 (.66–1.29)	.72 (.49–1.08)	1.31* (1.03–1.67)	1.06 (.85–1.35)	.85 (.67–1.08)	.66 (.39–1.12)	.72 (.46–1.13)	.89 (.60–1.32)
Physical & mental	.62 (.29–1.32)	.93 (.62–1.40)	1.17 (.88–1.55)	1.30 (.84–2.00)	1.88*** (1.43–2.48)	1.22 (.99–1.50)	2.16* (1.16–4.00)	2.03** (1.26–3.27)	1.31 (.95–1.81)
Physical									
Female	1.42*** (1.23–1.64)		1.27** (1.07–1.50)	1.57*** (1.41–1.74)	1.28*** (1.13–1.44)	1.24*** (1.11–1.39)			
Male									
North			1.23* (1.04–1.47)						
South									
Age 16–44	.77*** (.66–.90)		.79** (.66–.94)			1.13* (1.00–1.26)	2.18*** (1.79–2.65)	2.14*** (1.71–2.67)	1.65*** (1.37–2.00)
45–64									
No car						1.25** (1.09–1.42)	1.32* (1.02–1.72)		
Own car									
No computer								1.36** (1.08-1.71)	
Own computer									
Ethnic minority group				1.48*** (1.21–1.80)		1.47*** (1.20–1.81)			
Caucasian									
Not-working		1.63*** (1.37–1.94)			1.49*** (1.20-1.72)				
Working									
Not house owner House owner									
Intermediate job Managerial/professional									
Manual job									
Time				I = 1.22** (1.07–1.38)	K = 1.97*** (1.50–2.58)	I = 1.29*** (1.12–1.47)			

This table displays the odds ratios, thus the odds that the groups have utilized the relevant health or social care outcome compared to the reference group having utilized the relevant health or social care outcome. 95% confidence interval is displayed beneath in smaller print between brackets. Time variables I (1 in 2002, 2008, 2010), and  K (1 in 2010)

*p < .05, **p < .01, ***p < .001

**Table 3:** Secondary care of disabled individuals

	Outpatient			Daypatient			Inpatient		
	Limitations			Limitations			Limitations		
	None N = 11,308	Some N = 8552	Strong N = 6698	None N = 12,291	Some N = 8591	Strong N = 6702	None N = 12,172	Some N = 7738	Strong N = 6676
Mental	.72 (.51–1.01)	.60*** (.46–.79)	.53*** (.40–.70)	.76 (.53–1.09)	.56*** (.40–.78)	.64** (.47–.87)	1.03 (.70–1.51)	.76 (.56–1.05)	.72* (.54–.96)
Physical & mental	1.00 (.61–1.63)	1.02 (.78–1.33)	.96 (.78–1.19)	1.70* (1.02–2.83)	1.19 (.87–1.63)	1.14 (.90–1.45)	1.41 (.76–2.61)	.1.14 (.80–1.62)	1.16 (.92–1.47)
Physical									
Female	1.23*** (1.10–1.39)			1.17* (1.02–1.34)					
Male									
North/South									
Age 16–44	.88* (.78–.997)	.86* (.76–.97)	.79*** (.70–.89)		1.16* (1.01–1.34)	1.22** (1.07–1.40)			
45–64									
No car							1.42** (1.13–1.77)	1.32** (1.09–1.59)	1.18* (1.02–1.38)
Own car									
No computer					.85* (.73–.99)				
Own computer									
Ethnicity									
Not-working	1.20* (1.04–1.38)	1.23* (1.10–1.38)					1.38*** (1.15–1.66)	1.76*** (1.50–2.07)	1.39*** (1.21–1.60)
Working									
Not house owner	.79* (.64–97)				.78* (.64–.96)				
House owner									
Intermediate job	1.05 (.90–1.24)							1.06 (.87–1.29)	
Managerial/professional	1.31*** (1.15–1.50)							1.29** (1.07–1.56)	
Manual job									
Time	H = .63** (.45–.89)		H = .57** (.41–.80)						

This table displays the odds ratios, thus the odds that the groups have utilized the relevant health or social care outcome compared to the reference group having utilized the relevant health or social care outcome. 95% confidence interval is displayed beneath in smaller print. Time variables  H (1 in 2011)

*p < .05, **p < .01, ***p < .001

**Table 4:** Social care of disabled individuals

	In-work benefits			Extra-cost of living benefits			Out-of-work benefits		
	Limitations			Limitations			Limitations		
	None N = 9725	Some N = 6747	Strong N = 5950	None N = 9745	Some N = 7458	Strong N = 5301	None N = 9744	Some N = 6747	Strong N = 5301
Mental	1.73** (1.15–2.62)	.84 (.48–1.49)	1.43 (.81–2.54)	2.06** (1.27–3.33)	.76* (.57–.998)	.55*** (.40–.77)	5.14*** (3.31–7.98)	2.13*** (1.58–2.88)	1.48* (1.06–2.07)
Physical & mental	.45 (.14–1.45)	1.22 (.57–2.60)	1.12 (.57–2.19)	2.44* (1.14–5.24)	1.07 (.78–1.48)	1.01 (.79–1.30)	4.91*** (2.57–9.39)	3.59*** (2.28–5.67)	2.38*** (1.76–3.23)
Physical									
Female	2.12*** (1.64–2.72)	1.85*** (1.39–2.47)	2.84*** (1.95–4.13)					.42*** (.35–.50)	.37*** (.32–.44)
Male									
North	1.54*** (1.22–1.94)	1.37* (1.03–1.82)		1.45* (1.06–1.97)	1.33*** (1.15–1.55)	1.33*** (1.14–1.55)	1.45** (1.10–1.91)	1.27** (1.08–1.49)	1.26** (1.08–1.47)
South									
Age 16–44	3.40*** (2.63–4.41)	3.86*** (2.77–5.39)	2.40*** (1.70–3.40)			.79** (.66–.93)	2.15*** (1.60–2.90)		1.23* (1.03–1.48)
45–64									
No car	1.74*** (1.29–2.35)	1.86** (1.28–2.71)		1.54* (1.07–2.21)			3.48*** (2.57–4.72)	1.75*** (1.46–2.12)	1.83*** (1.53–2.20)
Own car									
No computer						.76*** (.65–.89)	1.71*** (1.26–2.33)	1.33** (1.11–1.60)	
Own computer									
Ethnic minority group	1.52* (1.00–2.30)								
Caucasian									
Not-working	.23*** (.14–.36)	.11*** (.06–.18)	.09*** (.05–.15)	7.46*** (5.43–10.26)	8.52*** (6.96–10.43)	8.76*** (7.08–10.83)	34.41*** (23.44–50.52)	49.54*** (37.05–66.25)	27.69*** (21.85–35.10)
Working part-time/full-time									
Not house owner	.38*** (.23–.63)	.33*** (.19–.58)	.41** (.21–.79)				.55* (.34–.89)	.64** (.48–.84)	.61*** (.47–.79)
House owner									
Intermediate job	.68** (.51–.91)	.67* (.47–.95)	.83 (.56–1.23)	.92 (.64–1.34)		.92 (.76–1.13)	.74 (.51–1.07)	.94 (.75–1.16)	.95 (.77–1.17)
Managerial/professional	.30*** (.22–.41)	.39*** (.27–.57)	.35*** (.23–.53)	.31*** (.20–.50)		.77** (.64–.93)	.40*** (.28–.59)	.61*** (.49–.77)	.74** (.61–.90)
Manual job									
Time	E = .10*** (.04–.25) F = 1.34* (1.07–1.69)	E = .10*** (.03–.32)	E = .09*** (.02–.36) F = 1.99*** (1.48–2.68)		C = 1.35** (1.13–1.61) D = 1.29* (1.06–1.57)	C = 1.37*** (1.14–1.65)	A = 2.37*** (1.63–3.46)	A = 1.25* (1.01–1.55) B = 1.26* (1.00–1.58)	A = 1.51*** (1.23–1.85)

This table displays the odds ratios, thus the odds that the groups have utilized the relevant health or social care outcome compared to the reference group having utilized the relevant health or social care outcome. 95% confidence intervals are displayed beneath in smaller print. Time variables A (1 from 2000 to 2005), B (1 in 2001), C (1 from 2006 to 2011), D (1 in 2004), and E (1 in 2000)

*p < .05, **p < .01, ***p < .001

Recent findings have shown that long-term unemployment is a problem among individuals with mental health problems. The models in which employment status was a significant predictor for health and social care utilisation were therefore repeated using the long-term unemployment variable. Results are displayed in supplementary material (Tables 1S–3S).

## Discussion

The British government implemented various policies and interventions in an effort to equalise access to health and social care. Unlike previous reports (Delgadillo et al. 2018; Dixon et al. 2007; Goddard and Smith 2001) the findings in this study do not reveal overwhelming evidence for horizontal inequity in health and social care utilisation based on socio-economic factors. This might be explained by the fact that our sample consisted solely of individuals with health problems and that we grouped our analyses based on care need. In prior studies (Morris et al. 2005), care need was found to be the strongest indicator of inequality.

Our findings did, however, indicate health and social care inequalities based on morbidity. Controlling for participant and socio-economic variables, our results brought to light that from 2000 to 2011 individuals with mental health problems were less likely to receive services from secondary health care providers and were more likely to receive out-of-work disability benefits compared to individuals with physical health problems. Significantly fewer individuals with mental health problems had full or part-time employment and significantly more individuals with mental health problems were long term unemployed compared to individuals with physical health problems. While many individuals with physical health problems were nudged into employment status transitions with the introduction of conditional criteria into UK’s welfare system, special employment programmes have been proven to be crucial to the vocational transition of individuals with mental health problems (Organisation for Economic Co-operation and Development 2003). Despite increasing evidence on the effectiveness of Independent Placement and Support to help individuals with mental health problems return and remain in the work force (Bond and Drake 2014), implementation of this vocational rehabilitation approach in England was limited during the duration of our study (Rinaldi et al. 2010). Hence, the British social care system was less successful to transition individuals with mental health problems compared to individuals with physical health problems from out-of-work benefits into paid employment.

### Policy and Clinical Implications

The findings of this study show that health care services provided by secondary care providers and social care services were not equally distributed based on morbidity. An explanation can be found in the five year forward view for mental health report written by the Mental Health Taskforce (2016). To use their quote “Mental health services have been underfunded for decades, and too many people have received no help at all, …..”. This was accentuated after the financial crisis of 2008 when the British National Health Service (NHS) made reductions in mental health care resources when similar reductions were not applied to physical health care resources (Docherty and Thornicroft 2015). Similar to our findings, the Mental Health Taskforce observed that 75% of the individuals with mental health problems receive no healthcare to deal with their symptoms. The taskforce further observed that too few individuals with mental health problems had access to the full range of interventions recommended by National Institute for Health and Care Excellence. This might explain why the individuals with mental health problems in this study were less likely than the individuals with physical health problems to receive secondary health care services. Since 2014 policy interventions have focused on increasing the funding for mental health services. These investments in mental health care did expand service provision, but it still needs to be evaluated if further equalisation of resources is needed (Baxter et al. 2018). Furthermore, mental health service utilisation is not only based on expressed need by the patient, but partially controlled by professionally defined need (Thomas et al. 2018; Levesque et al. 2013; Pilgrim 2012) as well as the political climate. It needs to be evaluated whether the current balance between patient’s expressed need, professionally defined need, and political climate is promoting a resource provision to mental health care that is equal to the resource provision to physical health care. Otherwise mental health policy amendments are recommended to adjust this balance to promote equalised resource provision between mental health and physical health care.

In the past two decades the government has reduced the collection of population health care data. This data is essential to evaluate the implications of health care legislation and policy, thus further health care policy recommendations include government investment in routine and repeated collection of population health data (Kilbourne et al. 2018). In addition, based on the findings, integration of vocational rehabilitation in mental health care is recommended to improve the individual with mental health problem’s integration in society.

### Limitations of the Study

Regretfully, details of expressed health care need were not assessed by the GHS/GLS survey nor was neglect of health care. Utilisation of primary health care, outpatient and emergency care services and day-patient and inpatient care services were measured, respectively, 2 weeks, 3 months and 1 year prior to the GHS/GLS survey. Due to these differences in measurement periods we could only determine that around 27% of the individuals with current mental health problems received health care services. This treatment rate is consistent with findings from the Adults Psychiatric Morbidity Survey 2007 (Cooper et al. 2010).

Participants are sampled from private households; individuals residing in institutions are not included. The results can therefore only be generalised to the individuals with health problems living in the community.

The British disability benefit system is part of an ever-changing complex social security system. Conditionality was first introduced to the disability benefits system during the time interval we studied, but conditionality became more stringent with the welfare reforms of 2013. Poor availability of national longitudinal data on individuals with mental health problems has made it difficult to track health and social care utilisation for individuals over time in Britain. Regretfully, the GLS data collection was discontinued in 2012, making it impossible to determine the impact of the more recent changes within the NHS (Docherty and Thornicroft 2015) and welfare system on health and social care utilisation or determine if increased political attention to mental health created parity of esteem.

## Conclusion

Our results revealed differences in labour market integration and health care utilisation between individuals with mental health problems and individuals with physical health problems. This disparity can be addressed by policy interventions. Health care should not only focus on providing medical treatment but also include vocational rehabilitation. Moreover, since population health and social care data has not been collected at regular intervals by the government, administrative data sources might inform policy makers if the implementation of interventions had its expected effect.

## Electronic supplementary material

Below is the link to the electronic supplementary material.

Supplementary file1 (DOC 109 kb)
