# Supplementary material for: Health and Social Care Diversity Among Individuals with Longstanding Physical and Psychological Health Problems: Pooled Repeated Cross Sectional Analyses
Source: Community Ment Health J. 2020 Feb 8;56(5):978–87. doi: 10.1007/s10597-020-00566-y (PMC7250952; doi:10.1007/s10597-020-00566-y)
Supplement: Supplementary file 1 — Supplementary file1 (DOC 109 kb) [file 10597_2020_566_MOESM1_ESM.doc]

Table 1S: Primary and Emergency care of disabled individuals

|  |  | **Nurse** | | |  | **General Practitioner** | | |  | **Emergency Care** | | |
| --- | --- | --- | --- | --- | --- | --- | --- | --- | --- | --- | --- | --- |
|  |  | *Limitations* | | |  | *Limitations* | | |  | *Limitations* | | |
|  |  | None | Some  N=5559 | Strong |  | None | Some  N=5384 | Strong |  | None | Some | Strong |
| Mental  Physical & Mental  *Physical* |  |  | .80  (.46-1.37)  .73  (.34-1.61) |  |  |  | 1.14  (.80-1.61)  2.08***  (1.41-3.07) |  |  |  |  |  |
| Female  *Male* |  |  |  |  |  |  | 1.30***  (1.11-1.51) |  |  |  |  |  |
| North/South England |  |  |  |  |  |  |  |  |  |  |  |  |
| Age |  |  |  |  |  |  |  |  |  |  |  |  |
| Own car |  |  |  |  |  |  |  |  |  |  |  |  |
| Own computer |  |  |  |  |  |  |  |  |  |  |  |  |
| Ethnicity |  |  |  |  |  |  |  |  |  |  |  |  |
| Not-working past 12M  *Working part-/fulltime* |  |  | 1.37*  (1.05-1.78) |  |  |  | 1.44***  (1.19-1.72) |  |  |  |  |  |
| House owner |  |  |  |  |  |  |  |  |  |  |  |  |
| Job level |  |  |  |  |  |  |  |  |  |  |  |  |
| Time |  |  |  |  |  |  | K=1.97***  (1.50-2.58) |  |  |  |  |  |

This table displays the odds ratios, thus the odds that the groups have utilized the relevant health or social care outcome compared to the reference group having utilized the relevant health or social care outcome. 95% confidence interval is displayed beneath in smaller print. Time variables K (1 in 2010) * p<.05 **p<.01 ***p<.001

Table 2S: Secondary care of disabled individuals

|  |  | **Outpatient** | | |  | **Daypatient** | | |  | **Inpatient** | | |
| --- | --- | --- | --- | --- | --- | --- | --- | --- | --- | --- | --- | --- |
|  |  | *Limitations* | | |  | *limitations* | | |  | *Limitations* | | |
|  |  | None | Some | Strong |  | None | Some | Strong |  | None | Some | Strong |
|  |  | N=9655 | N=5558 |  |  |  |  |  |  | N=10061 | N=5284 | n |
| Mental  Physical & Mental  *Physical* |  | .75  (.51-1.10)  .83  ( .45-1.54) | .60**  (.42-.87)  .88  (.59-1.33) |  |  |  |  |  |  | 1.07  (.67-1.71)  1.52  (.76-3.07) | .76  (.47-1.24)  .97  (.56-1.68) | .81  (.55-1.20)  1.24  (.87-1.77) |
| Female  *Male* |  | 1.25***  (1.10-1.42) |  |  |  |  |  |  |  |  |  |  |
| North/South England |  |  |  |  |  |  |  |  |  |  |  |  |
| Age 16-44  *45-64* |  | .87*  (.76-.99) | .94  (.81-1.09) |  |  |  |  |  |  |  |  |  |
| No car  *Own car* |  |  |  |  |  |  |  |  |  | 1.52**  (1.15-1.99) | 1.13  (.85-1.49) | 1.18  (.94-1.48) |
| *Own computer* |  |  |  |  |  |  |  |  |  |  |  |  |
| Ethnicity |  |  |  |  |  |  |  |  |  |  |  |  |
| Not-working past 12M  *Working part-time/fulltime* |  | 1.04  (.81-1.35) | 1.37***  (1.16-1.62) |  |  |  |  |  |  | 1.36*  (1.00-1.83) | 1.80***  (1.44-2.25) | 1.37**  (1.14-1.66) |
| Not house owner  *House owner* |  | .79*  (.63-.98) |  |  |  |  |  |  |  |  |  |  |
| Intermediate job  Managerial/professional  *Manual job* |  | 1.05  (.88-1.26)  1.33***  (1.16-1.54) |  |  |  |  |  |  |  |  | 1.21  (.94-1.55)  1.28*  (1.01-1.61) |  |
| Time |  | H=.63*  (.43-.92) |  |  |  |  |  |  |  |  |  |  |

This table displays the odds ratios, thus the odds that the groups have utilized the relevant health or social care outcome compared to the reference group having utilized the relevant health or social care outcome. 95% confidence interval is displayed beneath in smaller print. Time variables H (1 in 2011). * p<.05 **p<.01 ***p<.001

Table 3S: Social care of disabled individuals

|  |  | **In-work benefits** | | |  | **Extra-cost of living benefits** | | |  | **Out-of-work benefits** | | | |
| --- | --- | --- | --- | --- | --- | --- | --- | --- | --- | --- | --- | --- | --- |
|  |  | *Limitations* | | |  | *Limitations* | | |  | *Limitations* | | | |
|  |  | None  N=8294 | Some  N=4601 | Strong  N=3488 |  | None  N=8313 | Some  N=4850 | Strong  N=3146 |  | None  N=8312 | Some  N=4601 | | Strong  N=3146 |
| Mental  Physical & Mental  *Physical* |  | 1.91**  (1.25-2.92)  .56  (.17-1.81) | 1.10  (.60-2.00)  1.78  (.80-3.97) | 1.60  (.87-2.92)  1.16  (.56-2.42) |  | 1.28  (.52-3.14)  2.45  (.80-7.53) | .62  (.37-1.07)  1.28  (.80-2.06) | .60  (.35-1.03)  .83  (.53-1.30) |  | 6.14***  (3.21-11.73)  3.15  (.93-10.64) | 1.68  (.93-3.04)  4.85***  (2.19-10.77) | 1.10  (.63-1.90)  2.57***  (1.52-4.38) | |
| Female  *Male* |  | 2.11***  (1.63-2.74) | 1.74***  (1.29-2.36) | 2.68***  (1.82-3.96) |  |  |  |  |  |  | .46***  (.35-.62) | .43***  (.34-.55) | |
| North  *South* |  | 1.59***  (1.25-2.02) | 1.39*  (1.04-1.87) |  |  | 1.74*  (1.13-2.68) | 1.34*  (1.06-1.70) | 1.42**  (1.13-1.80) |  | 2.26***  (1.47-3.46) | 1.13  (.88-1.46) | 1.39**  (1.10-1.77) | |
| Age 16-44  *45-64* |  | 3.45***  (2.63-4.51) | 3.36***  (2.40-4.71) | 2.09***  (1.47-2.96) |  |  |  | .78*  (.60-.998) |  | 1.70*  (1.07-2.71) |  | .89  (.68-1.16) | |
| No car  *Own car* |  | 1.89***  (1.39-2.57) | 2.49***  (1.74-3.56) |  |  | 1.55  (.92-2.62) |  |  |  | 3.38***  (2.07-5.54) | 2.00***  (1.47-2.73) | 2.18***  (1.63-2.92) | |
| No computer  *Own computer* |  |  |  |  |  |  |  | .76*  (.60-.99) |  | 1.33  (.84-2.11) | 1.42*  (1.07-1.88) |  | |
| Ethnic minority group  *Caucasian* |  | 1.69*  (1.10-2.60) |  |  |  |  |  |  |  |  |  |  | |
| Not-working past 12M  *Working part-/fulltime* |  | .24**  (.10-.62) | .09***  (.03-.26) | .10***  (.03-.28) |  | 5.62***  (3.51-9.00) | 9.34***  (7.29-11.98) | 8.09***  (6.28-10.44) |  | 36.71***  (22.73-59.31) | 49.14***  (35.08-68.83) | 27.66***  (20.91-36.59) | |
| Not house owner  *House owner* |  | .37***  (.22-.63) | .31***  (.17-.55) | .38**  (.19-.76) |  |  |  |  |  | . 70  (.35-1.40) | .87  (..58-1.32) | .52**  (.35-.79) | |
| Intermediate job  Managerial/professional  *Manual job* |  | .69*  (.51-.92)  .29***  (.21-.40) | .69*  (.48-.98)  .41***  (.28-.61) | .73  (.48-1.10)  .34***  (.22-.52) |  | .88  (.54-1.45)  .22***  (.12-.43) |  | .88  (.65-1.19)  .79  (.59-1.05) |  | .68  (.39-1.19)  .47**  (.27-.83) | 1.07  (.77-1.50)  .72  (.51-1.02) | 1.04  (.77-1.41)  .67**  (.50-.89) | |
| Time |  | E=.10***  (.04-.25)  F=1.41**  (1.10-1.79) | E=.10***  (.03-.32) | E=.09***  (.02-.36)  F=2.07***  (1.51-2.83) |  |  | C=1.69***  (1.25-2.29)  D=1.49*  (1.03-2.15) | C=1.32  (.96-1.82) |  | A=1.67  (.91-3.05) | A=1.16  (.79-1.70)  B=1.24  (.93-1.64) | A=1.51*  (1.04-2.18) | |

This table displays the odds ratios, thus the odds that the groups have utilized the relevant health or social care outcome compared to the reference group having utilized the relevant health or social care outcome. 95% confidence intervals are displayed beneath in smaller print. Time variables A (1 from 2000 to 2005), B (1 in 2001), C (1 from 2006 to 2011), D (1 in 2004), and E (1 in 2000). * p<.05 **p<.01 ***p<.001
